# Home-based Aerobic Exercise and Resistance Training in Peritoneal Dialysis Patients: A Randomized Controlled Trial

**DOI:** 10.1038/s41598-019-39074-9

**Published:** 2019-02-22

**Authors:** Kiyotaka Uchiyama, Naoki Washida, Kohkichi Morimoto, Kaori Muraoka, Takahiro Kasai, Kentaro Yamaki, Kazutoshi Miyashita, Shu Wakino, Hiroshi Itoh

**Affiliations:** 1 Division of Endocrinology, Metabolism and Nephrology Department of Internal Medicine, Keio University School of Medicine, 35 Shinanomachi, Shinjuku-ku, Tokyo 160-8582 Japan; 2 Department of Nephrology, International University of Health and Welfare School of Medicine, 4-3, Kozunomori, Narita, Chiba 286-8686 Japan; 3 Department of Rehabilitation Medicine, Keio University School of Medicine, 35 Shinanomachi, Shinjuku-ku, Tokyo 160-8582 Japan

## Abstract

Potential effects of aerobic and resistance training in peritoneal dialysis (PD) patients have been partially elucidated. We investigated effects of a home-based exercise program on physical functioning and health-related quality of life (HRQOL) in PD patients. Patients were randomly assigned to exercise (n = 24) and usual care (n = 23) groups. The exercise patients performed aerobic exercise thrice weekly and resistance training twice weekly at home for 12 weeks. The usual care patients received no specific intervention. The distance in incremental shuttle walking test significantly improved in the exercise group compared with the usual care group (*P* = 0.02). Among the HRQOL subscales assessed using the Kidney Disease Quality of Life-Short Form questionnaire, kidney disease component summary (*P* = 0.03), physical role functioning (*P* = 0.01), emotional role functioning (*P* < 0.01), and role/social component summary (*P* < 0.01) significantly improved in the exercise group. Moreover, serum albumin was significantly maintained in the exercise group (*P* = 0.03). There were no reported adverse events associated with the intervention. To our knowledge, this is the first randomized controlled trial to indicate the beneficial effects of a 12-week home-based exercise program exclusively in PD patients.

## Introduction

Reduced physical functioning is common and progressive in chronic kidney disease (CKD) patients, especially those on chronic dialysis, and it is associated with high risks of morbidity and mortality^1–4^. Although several studies have confirmed the beneficial effects of exercise on aerobic capacity and health-related quality of life (HRQOL) in CKD and hemodialysis (HD) patients, studies involving peritoneal dialysis (PD) patients are limited, and no randomized controlled trial has exclusively included PD patients^5–8^. Because the cardiorespiratory response to exercise can possibly differ between HD and PD patients and PD patients can perform dialysis mainly at their homes, physical exercise programs performed under supervision during or between HD sessions in previous studies are not applicable to PD patients^6,9^.

Therefore, we designed a randomized controlled trial including only PD patients to clarify the effects of exercise in PD patients specifically. We adopted a home-based exercise program to increase patient acceptability of the program considering the lifestyle of PD patients performing dialysis at their homes.

## Results

### Patient Flow and Exercise Program Adherence

**Figure 1:**
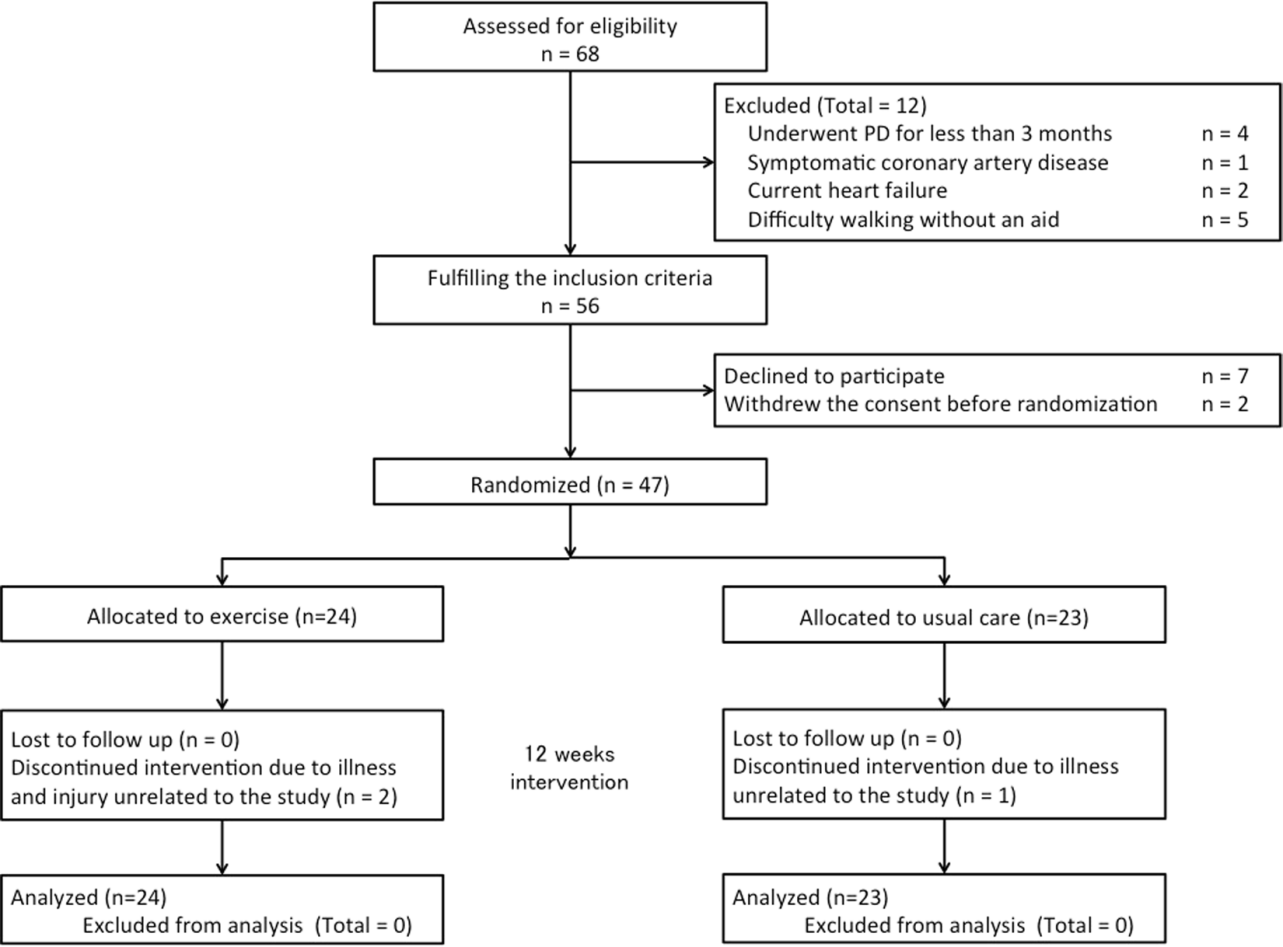
CONSORT diagram of the flow of patients through the various phases of the trial.

Among 68 PD outpatients assessed for eligibility during the study period, 56 fulfilled the inclusion criteria, and 47 patients were included in this study (see Fig. 1). Of these 47 patients, 24 were randomly assigned to the exercise group and 23 were randomly assigned to the usual care group.

Among the 24 patients in the exercise group, 1 discontinued the intervention because of hip fracture after a fall in the toilet (unrelated to exercise) and 1 discontinued the intervention because of severe cerebral infarction that caused paralysis on one side (unrelated to exercise). Among the 23 patients in the usual care group, 1 discontinued the study procedure because of the development of PD-related peritonitis, followed by catheter removal and transition to HD. In these patients who dropped out of the study, all data except physical functioning, HRQOL, and PD Kt/V data, were obtained 12 weeks after the baseline assessment. No patients were lost to follow-up.

Among the 22 patients in the exercise group who completed the intervention, the mean percentage adherence to prescribed aerobic exercise (AE) sessions was 52% ± 40% (interquartile range, 16–91) and the mean percentage adherence to prescribed resistance training (RT) sessions was 76% ± 37% (interquartile range, 69–100), according to data obtained from received postcards. Intervention-related serious adverse events were not noted.

### Baseline Characteristics

Table 1 summarizes the clinical characteristics of the study patients at the baseline. The exercise and usual care groups showed no difference in demographic, clinical, and biochemical characteristics; however, the proportion of patients with a smoking history tended to be higher in the usual care group than in the exercise group (*P* = 0.08). In contrast, nine patients who refused to participate were predominantly male (78%), with a mean age of 63.4 ± 15.4 years and PD vintage of 3.3 ± 2.1 years; none of these parameters were significantly different from those of the study participants in both groups.

**Table 1:** Demographic, clinical, and biochemical data of the study groups.

Variable	Usual care group (n = 23)	Exercise group (n = 24)	*P*-value
Age (years)	63.2 ± 9.5	64.9 ± 9.2	0.54
Sex (% male)	16 (70%)	19 (79%)	0.52
Diabetes mellitus	8 (35%)	6 (25%)	0.53
APD/CAPD	7/16 (30/70%)	6/18 (25/75%)	0.75
Cardiovascular disease	8 (35%)	7 (29%)	0.76
Smoking history	16 (70%)	7 (29%)	0.08
PD vintage (years)	4.0 ± 2.8	3.6 ± 2.7	0.59
Body weight (kg)	66.0 ± 13.6	61.0 ± 12.8	0.20
BMI (kg/m^2^)	24.6 ± 4.1	22.7 ± 3.5	0.11
Systolic blood pressure (mmHg)	137.5 ± 19.3	138.0 ± 22.1	0.90
Diastolic blood pressure (mmHg)	74.9 ± 13.8	76.0 ± 10.3	0.75
Urine output (mL/day)	534.8 ± 590.8	675.0 ± 716.6	0.47
Renal Kt/V	0.40 ± 0.49	0.44 ± 0.49	0.76
Ultrafiltration (mL/day)	1156.7 ± 624.5	1041.8 ± 513.3	0.49
PD Kt/V	1.38 ± 0.36	1.27 ± 0.48	0.38
Total Kt/V	1.78 ± 0.34	1.70 ± 0.44	0.44
Hemoglobin (g/dL)	10.6 ± 1.3	10.6 ± 1.3	0.90
Calcium (mmol/L)	9.5 ± 0.6	9.8 ± 2.2	0.57
Phosphorus (mmol/L)	5.5 ± 0.7	5.5 ± 1.5	0.90
PTH (pmol/L)	219.2 ± 200.7	207.3 ± 105.9	0.80

Abbreviations: APD, automated peritoneal dialysis; CAPD, continuous ambulatory peritoneal dialysis; PD, peritoneal dialysis; BMI, body mass index; PTH, parathyroid hormone.

### Effect of the Home-based Exercise on Physical Functioning

At the baseline, there were no significant differences in the incremental shuttle walking test (ISWT) (*P* = 0.83), handgrip strength (*P* = 0.91), and quadriceps strength (*P* = 0.43) between the exercise and usual care groups. After the intervention, the ISWT significantly improved (*P* = 0.02) in the exercise group when compared with the finding in the usual care group. However, there were no significant differences in handgrip strength (*P* = 0.13) and quadriceps strength (*P* = 0.55) (see Table 2). Similar findings were noted in the sensitivity analysis restricted to patients who completed the trial (see Table S1).

**Table 2:** Effects of the 12-week home-based exercise program on clinical outcomes (results of the paired *t*-test and 12-week ANCOVA).

	Usual care (n = 23)		*P*-value^a^	Exercise (n = 24)		*P*-value^a^	*P* for interaction^b^
	Baseline	Final		Baseline	Final		
Primary outcomes							
Aerobic capacity							
ISWT (m)	308.3 ± 109.6	282.2 ± 121.0	0.09	316.7 ± 149.9	341.7 ± 171.6	0.13	0.02
Secondary outcomes							
Muscle strength							
Handgrip strength (kg)	27.6 ± 7.1	25.7 ± 6.4	0.90	27.8 ± 5.5	27.3 ± 5.4	0.23	0.13
Quadriceps strength (kg)	24.5 ± 10.6	24.9 ± 10.5	0.75	22.2 ± 8.8	24.2 ± 10.7	0.27	0.55
Anthropometric data							
BMI (kg/m^2^)	24.6 ± 4.1	24.5 ± 4.3	0.56	22.7 ± 3.5	22.8 ± 3.4	0.43	0.38
Waist circumference (cm)	95.0 ± 11.8	96.5 ± 11.0	0.11	91.1 ± 9.4	92.9 ± 7.9	0.15	0.64
Leg circumference (cm)	36.9 ± 3.2	37.6 ± 3.4	0.08	35.4 ± 4.1	35.8 ± 3.8	0.37	0.35
SMI (kg/m^2^)	7.20 ± 1.12	7.38 ± 1.50	0.23	7.23 ± 1.27	7.19 ± 1.17	0.69	0.22
Biochemical analyses							
Albumin (g/L)	3.51 ± 0.44	3.37 ± 0.52	0.01	3.44 ± 0.49	3.48 ± 0.44	0.42	0.02
nPCR (g/kg/day)	0.88 ± 0.14	0.88 ± 0.19	0.85	0.93 ± 0.23	0.86 ± 0.22	0.13	0.34
Hemoglobin A1c (%)	5.64 ± 0.53	5.59 ± 0.59	0.60	5.87 ± 0.63	5.67 ± 0.66	<0.01	0.32
Total cholesterol (mmol/L)	182.3 ± 31.4	170.3 ± 31.4	0.02	176.8 ± 33.2	174.2 ± 34.7	0.52	0.16
HDL cholesterol (mg/dL)	47.0 ± 18.1	45.7 ± 14.2	0.52	57.8 ± 19.5	59.7 ± 20.8	0.37	0.07
Triglyceride (mg/dL)	115.6 ± 79.1	115.6 ± 89.4	0.90	164.7 ± 97.8	162.8 ± 89.5	0.37	0.53
HOMA-IR	2.54 ± 2.73	3.20 ± 5.42	0.61	2.89 ± 2.73	1.84 ± 1.66	0.04	0.23
Renal Kt/V	0.40 ± 0.49	0.36 ± 0.45	0.34	0.43 ± 0.49	0.37 ± 0.50	0.18	0.85
Ultrafiltration (mL/day)	1156.7 ± 624.5	1207.0 ± 500.2	0.61	1041.8 ± 513.2	1032.8 ± 501.6	0.76	0.30
PD Kt/V	1.38 ± 0.36	1.40 ± 0.37	0.81	1.27 ± 0.48	1.23 ± 0.38	0.32	0.11
CRP (mg/L)	0.27 ± 0.30	0.30 ± 0.50	0.81	0.22 ± 0.35	0.14 ± 0.25	0.20	0.19
hANP (pg/mL)	114.7 ± 101.7	117.3 ± 93.6	0.84	81.6 ± 51.5	79.5 ± 47.2	0.79	0.32
Arterial stiffness							
baPWV(m/s)	1.63 ± 0.30	1.71 ± 0.36	0.25	1.66 ± 0.37	1.63 ± 0.38	0.40	0.16

Abbreviations: ISWT, incremental shuttle walking test; BMI, body mass index; SMI, skeletal muscle mass index; nPCR, normalized protein catabolism rate; HDL, high-density lipoprotein; HOMA-IR, homeostasis model assessment of insulin resistance; CRP, C-reactive protein; hANP, human atrial natriuretic peptide; baPWV, brachial-ankle pulse wave velocity.

^a^Comparison between the baseline and final values.

^b^Comparison of the final values (values according to the final model with the baseline values as covariates).

**Figure 2:**
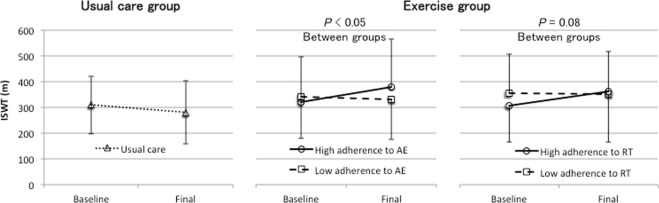
Dose–response relationship between the achieved incremental shuttle walking test (ISWT) across the usual care group and low adherence and high adherence to the home-based aerobic exercise (AE) or resistance training (RT) in the exercise group. The bars represent the standard deviations of the means. High and low adherence to AE were defined as adherence to >50% and ≤50% of the prescribed AE sessions, respectively, and high and low adherence to RT were defined as adherence to >86% and ≤86% of the prescribed RT sessions, respectively.

**Figure 3:**
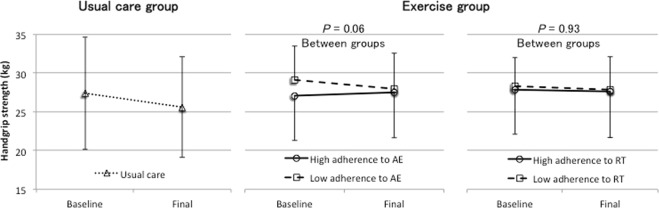
Dose–response relationship between the achieved handgrip strength across the usual care group and low adherence and high adherence to the home-based aerobic exercise (AE) or resistance training (RT) in the exercise group. The bars represent the standard deviations of the means. High and low adherence to AE were defined as adherence to >50% and ≤50% of the prescribed AE sessions, respectively, and high and low adherence to RT were defined as adherence to >86% and ≤86% of the prescribed RT sessions, respectively.

**Figure 4:**
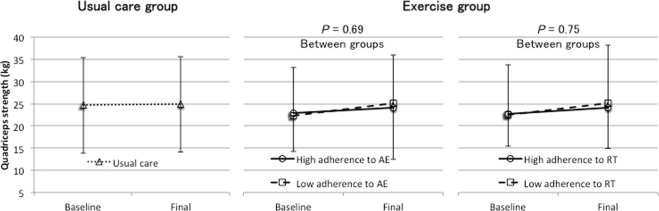
Dose–response relationship between the achieved quadriceps strength across the usual care group and low adherence and high adherence to the home-based aerobic exercise (AE) or resistance training (RT) in the exercise group. The bars represent the standard deviations of the means. High and low adherence to AE were defined as adherence to >50% and ≤50% of the prescribed AE sessions, respectively, and high and low adherence to RT were defined as adherence to >86% and ≤86% of the prescribed RT sessions, respectively.

Among the patients who completed the trial, we performed subgroup analyses to assess the dose–response relationship between adherence to the home-based exercise program and changes in physical functioning. The patients in the exercise group were divided into low and high AE/RT adherence groups according to the median value. The ISWT significantly improved (*P* < 0.05) and handgrip strength tended to be maintained (*P* = 0.06) in the high AE adherence group when compared with the findings in the low AE adherence group (see Figs 2–4). On the other hand, the ISWT tended to improve (*P* = 0.08) in the high RT adherence group when compared with the finding in the low RT adherence group. However, there were no significant differences in handgrip strength (*P* = 0.93) and quadriceps strength (*P* = 0.75) (see Figs 2–4).

### Effect of the Home-based Exercise on HRQOL

Table 3 summarizes the changes in the HRQOL scores. At the baseline, only the subscale of social functioning was significantly lower in the exercise group than in the usual care group (*P* < 0.01). After the intervention, among the domains in Kidney Disease Quality of Life (KDQOL), kidney disease component summary (KDCS) significantly improved (*P* = 0.03) in the exercise group when compared with the finding in the usual care group. The score for the effect of kidney disease was significantly higher at the final assessment than at the baseline in the exercise group (*P* = 0.04). Among the domains in 36-Item Short Form Health Survey (SF-36), physical role functioning, emotional role functioning, and role/social component summary (RCS) significantly improved (*P* = 0.01, *P* = 0.005, and *P* < 0.01, respectively) and bodily pain tended to improve (*P* = 0.07) in the exercise group when compared with the findings in the usual care group. The score for vitality was significantly higher at the final assessment than at the baseline in the exercise group (*P* < 0.05). In the sensitivity analysis restricted to patients who completed the trial, similar findings were noted; however, the score for vitality tended to improve (*P* = 0.06) in the exercise group when compared with the findings in the usual care group (see Table S2).

**Table 3:** Effects of the 12-week home-based exercise program on health-related quality of life scores (results of the paired *t*-test and 12-week ANCOVA).

	Usual care (n = 23)		*P*-value^a^	Exercise (n = 24)		*P*-value^a^	*P* for interaction^b^
	Baseline	Final		Baseline	Final		
KDQOL							
Symptoms/problems	77.4 ± 14.3	78.7 ± 15.2	0.60	78.2 ± 11.5	79.5 ± 11.4	0.54	0.90
Effects of kidney disease	77.6 ± 14.4	78.1 ± 15.6	0.83	76.1 ± 15.0	79.8 ± 14.3	0.04	0.22
Burden of kidney disease	47.0 ± 20.1	42.4 ± 19.2	0.09	40.6 ± 22.6	48.1 ± 21.7	0.17	0.11
Cognitive function	92.4 ± 9.1	92.4 ± 9.5	0.90	90.3 ± 11.1	90.3 ± 10.7	0.9	0.75
Quality of social interaction	92.3 ± 11.2	88.1 ± 14.9	0.14	85.1 ± 15.8	88.4 ± 10.6	0.21	0.24
Sleep	65.0 ± 16.7	60.9 ± 18.1	0.17	57.4 ± 18.2	56.6 ± 16.7	0.82	0.90
Social support	85.5 ± 18.3	81.0 ± 16.9	0.18	75.0 ± 22.0	80.7 ± 18.7	0.36	0.76
Dialysis staff encouragement	79.6 ± 24.4	83.0 ± 17.4	0.51	84.5 ± 14.2	80.6 ± 21.7	0.48	0.50
Patient satisfaction	81.7 ± 15.7	78.0 ± 22.8	0.56	79.2 ± 17.2	75.2 ± 17.6	0.43	0.82
KDCS	75.8 ± 10.7	72.5 ± 10.0	0.05	72.4 ± 9.0	74.3 ± 10.2	0.15	0.03
SF-36							
Physical functioning	77.4 ± 14.4	73.2 ± 13.9	0.13	73.3 ± 22.3	76.0 ± 15.7	0.43	0.15
Physical role functioning	73.4 ± 23.3	62.2 ± 26.9	0.04	64.6 ± 23.6	71.9 ± 22.0	0.06	0.01
Bodily pain	77.7 ± 22.5	67.5 ± 24.4	0.05	65.2 ± 24.2	73.0 ± 19.1	0.1	0.06
General health	48.0 ± 15.8	45.7 ± 17.4	0.42	43.0 ± 19.1	43.7 ± 17.9	0.42	0.61
Vitality	57.9 ± 21.0	54.8 ± 20.3	0.43	51.6 ± 21.6	57.5 ± 20.3	<0.05	0.12
Social functioning	80.4 ± 24.1	74.3 ± 26.0	0.21	59.9 ± 27.6	71.8 ± 19.6	0.06	0.47
Emotional role functioning	81.9 ± 22.0	64.3 ± 31.8	0.02	73.6 ± 21.1	77.5 ± 19.4	0.4	<0.01
Mental health	73.7 ± 19.4	73.2 ± 17.6	0.88	69.2 ± 17.4	71.5 ± 18.8	0.38	0.74
PCS	41.3 ± 9.7	38.2 ± 9.2	1.00	40.1 ± 14.9	41.0 ± 8.1	0.71	0.12
MCS	51.2 ± 9.3	52.6 ± 9.0	0.50	48.7 ± 8.8	49.8 ± 9.6	0.42	0.57
RCS	47.8 ± 12.4	40.6 ± 15.4	0.01	41.0 ± 11.6	45.3 ± 11.1	0.06	<0.01

Abbreviations: KDQOL, Kidney Disease Quality of Life; SF-36, Medical Outcomes Study 36-Item Short-Form Health Survey MOS; KDCS, kidney disease component summary; PCS, physical component summary; MCS, mental component summary; RCS, role/social component summary.

^a^Comparison between the baseline and final values.

^b^Comparison of the final values (values according to the final model with the baseline values as covariates).

### Effect of the Home-based Exercise on Anthropometric Data, Biochemical Analyses and Arterial Stiffness

Anthropometric data were not significantly different between the final assessment and baseline in both groups, and there were no significant differences between the groups (see Table 2). On the other hand, serum albumin was significantly maintained (*P* = 0.02) and high-density lipoprotein (HDL) cholesterol tended to improve (*P* = 0.07) in the exercise group when compared with the findings in the usual care group. Hemoglobin A1c and homeostasis model assessment of insulin resistance (HOMA-IR) were significantly lower at the final assessment than at the baseline in the exercise group (*P* < 0.01 and *P* = 0.04, respectively); however, when compared with the finding in the usual care group, the improvement was not significant. For other parameters, including renal Kt/V, CRP, and human atrial natriuretic peptide (hANP), there were no significant differences in the changes between the two groups.

The change in brachial-ankle pulse wave velocity (baPWV) was not significantly different between the two groups. In sensitivity analysis restricted to patients who completed the trial, similar findings were noted (see Table S1). Blood pressure, hemoglobin levels, and parameters of CKD–mineral bone disorder were well maintained, and there were no significant differences between the groups (data not shown).

## Discussion

We noted improvements in aerobic capacity assessed with the ISWT, several domains of HRQOL, and the serum albumin level in the exercise group when compared with the findings in the usual care group.

Impaired physical capacity is prevalent in CKD patients, especially those on dialysis, and its etiology is multifactorial and remains unclear^2^. Exercise capacity has been assessed in patients on HD, which is the major approach of renal replacement therapy globally^10^; however, exercise capacity has been partially described in PD patients, which is a less frequent approach. Few studies have reported on the prevalence rates and predictors of reduced exercise capacity in PD patients and on the association of physical functioning with all-cause mortality and technical failure in PD patients^11,12^. The findings of these studies indicate that an exercise program to improve physical functioning will most likely have beneficial effects in PD patients.

In predialysis CKD patients, many previous randomized controlled trials revealed a significant or insignificant improvement in peak heart rate oxygen uptake (VO_2peak_) after interventions (AE or AE with RT)^5,13–17^. Additionally, improvements in various domains of HRQOL, as well as the maintenance of renal function and decrease of PWV have been reported^5,13,15,18,19^. Many randomized controlled trials involving dialysis patients revealed a significant improvement in VO_2peak_ or the 6-min walk test and the HRQOL^6,8,20–22^. However, few randomized controlled trials involving PD patients have been published^8,23^, and a small matched cohort study including only PD patients has been reported^7^. This is partly because of the lower number of PD patients compared with that of HD patients and the lifestyle of PD patients involving the conduction of dialysis at home^10^. To the best of our knowledge, our study is the first randomized controlled trial on exercise training that exclusively recruited PD patients. Although adherence to our home-based program was not high, we observed many statistical differences in the intention-to-treat analysis, and a dose–response relationship was noted, especially regarding adherence to AE.

The novelty of our study includes the use of the ISWT as aerobic capacity. Although the VO_2peak_ measured by general cardio-pulmonary exercise test is the gold standard approach, the ISWT is a more objective approach than the 6-min walk test, and its utility has been well described in patients with chronic obstructive pulmonary disease or heart failure. In addition, it is being increasingly validated in predialysis CKD, HD, and PD patients^24–27^, and a strong correlation between the ISWT and VO_2peak_ in predialysis CKD patients was reported^28^. We also previously demonstrated positive relationship between the ISWT and various subscales of HRQOL in PD patients^29^. Moreover, the simplicity and less invasiveness of the ISWT when compared with the cardio-pulmonary exercise test might increase the patient recruitment rate and maximize the statistical power and generalizability in our small number of PD patients. Another advantage was that we could provide instructions for AE directly from the results of the ISWT. This approach cannot be adopted for the cardio-pulmonary exercise test involving a treadmill.

This trial has several limitations. First, body mass index (BMI) and skeletal muscle mass index (SMI) are highly influenced by the volume status in PD patients, in whom systemic fluid overload is commonly observed^30^. We examined hANP as a marker of the hydration status and found that its change did not differ between the study groups. However, bioimpedance analysis is a better method to evaluate the volume status. Second, adherence to the home-based exercise program was not very high, despite sending a postcard weekly to patients in the exercise group to check adherence. Although the proportion of recruited patients was high (approximately 84%), limited adherence was mostly because of the unsupervised nature of the home-based exercise program and partly because of issues with instructions for both AE and RT. In addition, our method of sending a post card weekly to the patients might be not effective to improve the adherence of the patients. As reported in previous studies, direct contacts or phone calls with encouragement and feedback^5,6,8,16^ as well as setting short-term supervised training period preceding home-based exercise period^13^, which is totally dependent on the patients, might be helpful to achieve higher adherence rate. Finally, since the current trial had a short follow-up period, it remains unclear whether exercise can improve mortality or technical survival in PD patients. To prove the benefit of exercise on these critical hard outcomes, future trials with longer-term randomization were necessary.

In conclusion, this is the first study to indicate the beneficial effects of a 12-week home-based exercise program involving AE and RT in PD patients. The program can improve aerobic capacity, various subscales of HRQOL, and some nutritional and metabolic parameters without any adverse effects.

## Methods

### Study Population

Stable PD patients aged 20–90 years who had started with and undergone PD for at least 3 months at Keio University Hospital in Tokyo, Japan, between November 2016 and March 2018 were evaluated for inclusion in this randomized controlled trial. Sample size calculation was based on the findings of a previous matched cohort study that recruited only PD patients, which assessed VO_2peak_ as an aerobic capacity, because no exercise intervention studies assessing ISWT among PD patients are available and minimum clinically important difference in terms of ISWT has not been suggested in PD patients^7^. We revealed that 30 patients per study group were required to detect a 20% improvement in aerobic capacity (unpaired Student’s *t*-test; β = 0.20; α = 0.05), and adjusting this for the analysis of covariance (ANCOVA) under an assumed pre–post correlation of approximately 0.6 yields a target sample size of 38^31^. Further, inflating this for an estimated attrition rate of 20%, we calculated an overall sample size as 48. Considering this number and the small number of PD patients at our clinic, we assessed all PD patients for eligibility. The exclusion criteria were as follows: uncontrolled hypertension (blood pressure >180/110 mmHg), severe anemia (hemoglobin level < 7 mg/dL), active and proliferative diabetic retinopathy, symptomatic coronary artery disease or cerebrovascular disease within 3 months before study recruitment, current heart failure (New York Heart Association classes III and IV), symptomatic and fatal arrhythmia, significant valvular heart disease, and difficulty walking without a walking aid owing to orthopedic problems, a history of cerebrovascular disease, or a history of peripheral artery disease.

### Study Design and Randomization

The ethics committee of Keio University Hospital reviewed and approved the protocol of this randomized, single-blind, parallel trial (approval number: 20160202), and written informed consent was obtained from all participants. The trial was registered in a public trial registry (UMIN-CTR number: UMIN0000024907; 21/11/2016; https://upload.umin.ac.jp/cgi-open-bin/ctr_e/ctr_view.cgi?recptno=R000027068), and it was conducted in adherence with the Declaration of Helsinki and Consolidated Standards of Reporting Trials (CONSORT).

After the baseline assessment, block randomization with a block size of four was performed by an individual not associated with the trial, using computer-generated random numbers. The patients were equally allocated into control and exercise groups. Regardless of the assignment, all patients visited PD clinic monthly. Blinding patients or the rehabilitation doctors to group assignment was impossible; however, the nephrologists responsible for the PD clinics were blinded. The patients in the control group received usual care and advice to maintain their lifestyles. The patients in the exercise group were instructed to perform individualized AE and RT at home by the same rehabilitation doctor.

### Outcome Measures

All outcome measures were assessed at the baseline and 12 weeks after the program in all available patients, regardless of adherence to the home-based exercise program. The primary outcomes were the ISWT and HRQOL, and the secondary outcomes were muscle strength, anthropometric and biochemical data related to PD, and arterial stiffness.

#### Physical Functioning

Aerobic capacity was measured with the ISWT, which requires patients to walk between two cones spaced 10 m apart, with the pace set by a beeper. The speed of the beeper increased gradually until the patient could not keep up with the set pace or until they stopped owing to fatigue. The total distance was measured and used for the analysis.

Handgrip strength was assessed in both hands using a dynamometer in the standing position with both arms naturally placed down, according to the instructions of the Japan Sports Agency^32^. Handgrip strength was measured twice in both hands, and the highest value was used for the analysis. The quadriceps strength was assessed in both legs using an isokinetic dynamometer (μTasF-1, Anima, Tokyo, Japan), with a specific reading of isometric peak torque. The quadriceps strength was measured twice in both legs, and the maximum peak torque was used for analysis.

#### HRQOL

HRQOL was measured using the KDQOL-SF Japanese version 1.3 with SF-36v2 Japanese version^33,34^, which includes subscales on QOL specific to kidney disease and dialysis (KDQOL) and on general HRQOL (SF-36). The kidney disease-specific subscales were averaged to derive KDCS. The following three summary scales were calculated from scores in SF-36: physical component summary (PCS), mental component summary (MCS), and RCS^35^.

#### Anthropometric Data and Biochemical Analyses

BMI (kg/m^2^) was calculated from height (cm) and weight (kg). Waist circumference (cm) at the level of the umbilicus and the mean of circumferences of both legs (cm) at the level of the thigh midline were calculated. Whole-body dual-energy X-ray absorptiometry (DXA; QDR 4500/A, Hologic, Waltham, MA, USA) was used to assess body composition, and SMI was calculated as appendicular lean mass divided by the square of height (kg/m^2^).

Blood, urine, and PD fluid samples were obtained from the patients for biochemical analyses. The serum albumin level (mg/dL) and normalized protein catabolism rate (g/kg/day) were measured as nutritional markers. Hemoglobin A1c (%), total cholesterol (mg/dL), HDL cholesterol (mg/dL), and triglyceride (mg/dL) levels were measured as metabolic parameters. Moreover, HOMA-IR was calculated from the fasting blood glucose (mg/dL) and fasting insulin (μU/mL) levels. Renal Kt/V, ultrafiltration volume (mL/day), and PD Kt/V were measured as PD-related parameters. The C-reactive protein level (mg/L) was measured as an inflammation marker, and hANP level (pg/mL) was measured to assess fluid status.

The prescriptions of antihypertensive drugs, phosphate-lowering agents, and erythropoiesis-stimulating agents could be adjusted by the nephrologist. However, the prescriptions of the PD dialysate, antidiabetic drugs, and antilipemic agents were not changed during the study period in both the usual care and exercise groups.

#### Arterial Stiffness

We measured baPWV using a cardiovascular screening device (BP-203RPEIII, Omron Healthcare, Kyoto, Japan) after 5 min of rest in the supine position. Volume waveforms were obtained for the brachial and tibial arteries simultaneously, and the transmission time from the right brachial pulse to each ankle pulse was determined. The pressure waveform was measured for an average of 10 s twice^36^. The transmission distance from the arm to each ankle was estimated according to body height, and the baPWV value was calculated as the transmission distance divided by the transmission time. The mean bilateral baPWV value was used for the analysis.

### Exercise Intervention

Regarding AE, patients in the exercise group were asked to perform unsupervised walking thrice weekly for 12 weeks. The target training zone was set at 40–60% of the peak heart rate, as determined in the baseline ISWT, with a rating of 11–13 on the Borg Rating of Perceived Exertion scale. The target walking speed was almost the same as the speed two levels below the maximum speed in the ISWT, and patients were trained to walk at the target speed under the supervision of the rehabilitation doctor for 50 m or more at the baseline examination. Moreover, they were requested to start and increase the walking intensity according to their capabilities. Investigators encouraged the patients to start the program at 20 min/session and progress to 30 min/session.

RT was prescribed at 70% of one repetition maximum (RM). One RM is the maximum amount of weight an individual can lift or press only once, and the target training weight was almost the same as the weight an individual could lift or press 10 times. Patients were instructed to train a variety of upper and lower body muscle groups (e.g., latissimus, deltoid, biceps, quadriceps, and gastrocnemius muscles) using Theraband (Hygenic Corp., Akron, OH, USA) for 1 set of 10 repetitions twice weekly. One RM was reassessed monthly along with a visit to PD clinic, and the program was adjusted accordingly.

The patients were sent a postcard weekly to monitor their adherence to both AE (including the duration of each walking session) and RT. The number of sessions performed in 12 weeks was calculated as a percentage of the total possible sessions. These tasks were performed by the same rehabilitation doctor.

### Statistical analysis

The normal distribution of variables was verified by the Kolgomorov–Smirnov test. Data are summarized as means ± standard deviations or percentages. The unpaired Student’s *t*-test (continuous variables) and chi-squared test (binary variables) were used for comparisons between the patient groups, and the paired *t*-test was used to compare within-group changes over time. ANCOVA, with the baseline values as covariates and 12-week values as dependent variables, was used to test for significant differences between the exercise and usual care groups or between the high adherence and low adherence groups.

We used intention-to-treat analyses for all outcomes. In three patients who did not participate throughout the study, complete data on physical functioning, HRQOL, and parameters related to PD fluid were not obtained; therefore, multiple imputations were performed to impute lost data values automatically. Five imputations were performed, and the average was calculated. Sensitivity analyses were performed with per-protocol analyses. Interim analyses were not performed.

SPSS software for Mac (ver. 25; IBM Corp., Armonk, NY, USA) was used to perform all analyses. A *P*-value < 0.05 was considered statistically significant.

## Supplementary information

Supplemental Table

## Data Availability

The datasets generated during and/or analyzed during the current study are available from the corresponding author on reasonable request.
