# Supplementary material for: Home-based Aerobic Exercise and Resistance Training in Peritoneal Dialysis Patients: A Randomized Controlled Trial
Source: Sci Rep. 2019 Feb 22;9:2632. doi: 10.1038/s41598-019-39074-9 (PMC6385506; doi:10.1038/s41598-019-39074-9)
Supplement: Supplementary file 1 — Supplemental Table [file 41598_2019_39074_MOESM1_ESM.pdf]

Home-based Aerobic Exercise and Resistance Training in Peritoneal Dialysis Patients: A Randomized Controlled Trial

Kiyotaka Uchiyama, MD,[1] Naoki Washida, MD, PhD,[1, 2] Kohkichi Morimoto, MD, PhD,[1] Kaori Muraoka, MD, PhD,[3] Takahiro Kasai, MD,[2] Kentaro Yamaki, MD,[1] Kazutoshi Miyashita, MD, PhD,[1] Shu Wakino, MD, PhD,[1] Hiroshi Itoh, MD, PhD[1]

[1]Division of Endocrinology, Metabolism and Nephrology Department of Internal Medicine, Keio University School of Medicine, 35 Shinanomachi, Shinjuku-ku, Tokyo 160-8582, Japan

[2]Department of Nephrology, International University of Health and Welfare School of Medicine, 4-3, Kozunomori, Narita, Chiba 286-8686, Japan

[3]Department of Rehabilitation Medicine, Keio University School of Medicine, 35 Shinanomachi, Shinjuku-ku, Tokyo 160-8582, Japan

Supplemental Material File Listing:

Supplemental Table 1, Supplemental Table 2

Corresponding author:

Kiyotaka Uchiyama, MD

35 Shinanomachi, Shinjuku-ku, Tokyo 160-8582, Japan

Tel: +81-3-5363-3796; Fax: +81-3-3359-2745; E-mail: kiyo.0817.piyo@keio.jp

Supplemental Table 1. Effects of the 12-week home-based exercise program on clinical outcomes: Per-protocol analysis in patients who completed the trial (results of the paired *t*-test and 12-week ANCOVA)

	**Usual care (n = 22)**			**Exercise (n = 22)**			***P* for**
	**Baseline**	**Final**	***P*-value[a]**	**Baseline**	**Final**	***P*-value[a]**	**interaction[b]**
Primary outcomes							
Aerobic capacity							
ISWT (m)	308.3 ± 109.6	282.2 ± 121.0	0.07	330.5 ± 145.5	355.6 ± 168.0	0.16	0.02
Secondary outcomes							
Muscle strength							
Handgrip strength (kg)	27.4 ± 7.2	25.6 ± 6.5	0.9	28.1 ± 5.1	27.7 ± 5.2	0.37	0.1
Quadriceps strength (kg)	24.7 ± 10.8	24.9 ± 10.8	0.86	22.6 ± 9.1	24.7 ± 11.0	0.28	0.49
Anthropometric data							
BMI (kg/m^2)	24.8 ± 4.0	24.8 ± 4.1	0.83	22.9 ± 3.6	23.0 ± 3.5	0.55	0.69
Waist circumference (cm)	95.0 ± 11.8	96.5 ± 11.0	0.11	91.1 ± 9.4	92.9 ± 7.9	0.15	0.64
Leg circumference (cm)	37.1 ± 3.2	37.7 ± 3.4	0.14	35.8 ± 4.0	36.1 ± 3.7	0.49	0.46
SMI (kg/m^2)	7.26 ± 1.09	7.45 ± 1.49	0.23	7.34 ± 1.27	7.28 ± 1.19	0.59	0.2

Biochemical analyses							
Albumin (g/L)	3.55 ± 0.41	3.42 ± 0.41	0.03	3.46 ± 0.49	3.51 ± 0.44	0.37	0.03
nPCR (g/kg/day)	0.88 ± 0.14	0.87 ± 0.18	0.49	0.93 ± 0.24	0.86 ± 0.22	0.15	0.48
HbA1c (%)	5.66 ± 0.54	5.61 ± 0.60	0.63	5.79 ± 0.58	5.55 ± 0.55	0.001	0.18
Total cholesterol (mmol/L)	182.7 ± 32.0	171.7 ± 31.4	0.03	176.3 ± 34.2	172.0 ± 33.8	0.3	0.36
HDL cholesterol (mg/dL)	47.1 ± 18.6	46.0 ± 14.5	0.58	56.6 ± 19.3	58.1 ± 18.6	0.49	0.07
Triglyceride (mg/dL)	168.7 ± 98.2	166.7 ± 89.6	0.9	119.3 ± 81.7	117.4 ± 92.2	0.9	0.48
HOMA-IR	2.64 ± 2.76	3.32 ± 5.52	0.61	2.92 ± 2.82	1.91 ± 1.72	0.07	0.25
Renal Kt/V	0.42 ± 0.50	0.36 ± 0.46	0.01	0.49 ± 0.50	0.41 ± 0.50	0.19	0.69
Ultrafiltration (mL/day)	1163.8 ± 638.2	1193.2 ± 507.5	0.77	1029.7 ± 519.1	1025.5 ± 502.2	0.9	0.25
PD Kt/V	1.36 ± 0.35	1.38 ± 0.35	0.76	1.24 ± 0.49	1.19 ± 0.38	0.37	0.13
CRP (mg/L)	0.27 ± 0.53	0.21 ± 0.26	0.59	0.23 ± 0.36	0.12 ± 0.23	<0.05	0.19
hANP (pg/mL)	105.9 ± 94.8	108.7 ± 85.9	0.84	82.3 ± 53.6	85.2 ± 56.0	0.69	0.54
Arterial stiffness							
baPWV (m/s)	1.61 ± 0.29	1.69 ± 0.35	0.25	1.66 ± 0.39	1.65 ± 0.37	0.81	0.29

Abbreviations: ISWT, incremental shuttle walking test; BMI, body mass index; SMI, skeletal muscle mass index; nPCR, normalized protein catabolism rate; HDL, high-density lipoprotein; HOMA-IR, homeostasis model assessment of insulin resistance; CRP, C-reactive protein; hANP, human atrial natriuretic peptide; baPWV, brachial–ankle pulse wave velocity

[a]Comparisons between the baseline and final values

[b]Comparisons of the final values (values according to the final model with the baseline values as covariates)

Supplemental Table 2. Effects of the 12-week home-based exercise program on health-related quality of life scores: Per-protocol analysis in patients who completed the trial (results of the paired *t*-test and 12-week ANCOVA)

	**Usual care (n = 22)**			**Exercise (n = 22)**			***P* for**
	**Baseline**	**Final**	***P*-value[a]**	**Baseline**	**Final**	***P*-value[a]**	**interaction[b]**
KDQOL							
Symptoms/problems	77.0 ± 14.5	78.8 ± 15.6	0.46	79.5 ± 10.5	79.5 ± 11.7	0.99	0.79
Effects of kidney disease	77.3 ± 14.7	78.0 ± 15.9	0.79	77.0 ± 15.3	80.8 ± 15.3	0.24	0.45
Burden of kidney disease	48.0 ± 20.0	42.3 ± 19.7	0.08	42.3 ± 22.8	48.2 ± 23.0	0.31	0.13
Cognitive function	92.4 ± 9.1	92.4 ± 9.7	0.9	90.9 ± 11.0	89.3 ± 11.6	0.28	0.38
Quality of social interaction	92.0 ± 11.4	88.2 ± 15.2	0.19	86.4 ± 14.1	88.5 ± 11.3	0.33	0.25
Sleep	65.1 ± 17.0	61.0 ± 18.5	0.19	59.4 ± 17.5	58.9 ± 16.5	0.89	0.75
Social support	84.8 ± 18.5	81.1 ± 17.3	0.26	76.5 ± 22.2	79.8 ± 20.4	0.6	0.86
Dialysis staff encouragement	79.6 ± 24.4	82.9 ± 19.2	0.9	84.5 ± 14.2	80.6 ± 23.8	0.58	0.78
Patient satisfaction	81.7 ± 15.7	78.1 ± 23.9	0.35	79.5 ± 17.8	76.5 ± 19.0	0.41	0.91
KDCS	75.6 ± 10.8	72.5 ± 10.2	0.08	73.1 ± 9.2	74.5 ± 10.6	0.27	0.06
SF-36							

Physical functioning	77.0 ± 14.7	73.2 ± 14.2	0.18	75.0 ± 22.1	75.7 ± 18.2	0.82	0.29
Physical role functioning	72.2 ± 23.1	61.9 ± 27.5	0.07	65.1 ± 24.4	74.7 ± 23.1	0.05	0.01
Bodily pain	76.6 ± 22.5	67.4 ± 25.0	0.08	65.6 ± 24.5	73.6 ± 19.4	0.1	0.07
General health	48.3 ± 16.2	45.7 ± 17.8	0.39	44.4 ± 19.4	43.8 ± 18.6	0.81	0.76
Vitality	59.7 ± 19.6	54.8 ± 20.8	0.21	54.3 ± 20.4	59.3 ± 20.0	0.08	0.06
Social functioning	80.7 ± 24.6	74.4 ± 26.6	0.22	59.7 ± 28.8	71.0 ± 20.9	0.11	0.57
Emotional role functioning	81.9 ± 22.0	64.0 ± 32.6	<0.01	73.9 ± 22.0	79.5 ± 20.5	0.28	<0.01
Mental health	73.9 ± 19.8	73.2 ± 18.0	0.86	70.3 ± 17.8	71.8 ± 19.4	0.57	0.81
PCS	40.8 ± 9.6	38.1 ± 9.4	0.12	41.0 ± 14.9	41.0 ± 8.8	0.87	0.19
MCS	51.2 ± 9.3	52.7 ± 9.2	0.71	49.5 ± 8.6	49.9 ± 9.9	0.77	0.61
RCS	47.3 ± 12.4	40.4 ± 15.7	0.02	40.5 ± 12.0	46.2 ± 12.0	0.03	<0.01

Abbreviations: KDQOL, Kidney Disease Quality of Life; SF-36, Medical Outcomes Study 36-Item Short-Form Health Survey MOS; KDCS, Kidney Disease Component Summary; PCS, physical component summary; MCS, mental component summary; RCS, role/social component summary

[a]Comparisons between the baseline and final values

[b]Comparisons of the final values (values according to the final model with the baseline values as covariates)
